# A field-based indicator for determining the likelihood of *Ixodes scapularis* establishment at sites in Ontario, Canada

**DOI:** 10.1371/journal.pone.0193524

**Published:** 2018-02-27

**Authors:** Katie M. Clow, Nicholas H. Ogden, L. Robbin Lindsay, Curtis B. Russell, Pascal Michel, David L. Pearl, Claire M. Jardine

**Affiliations:** 1 Department of Pathobiology, Ontario Veterinary College, University of Guelph, Guelph, Ontario, Canada; 2 National Microbiology Laboratory, Public Health Agency of Canada, Saint-Hyacinthe, Quebec, Canada; 3 National Microbiology Laboratory, Public Health Agency of Canada, Winnipeg, Manitoba, Canada; 4 Enteric, Zoonotic and Vector-Borne Diseases, Communicable and Infectious Disease Prevention and Control, Public Health Ontario, Toronto, Ontario, Canada; 5 Office of the Chief Science Officer, Public Health Agency of Canada, Ottawa, Ontario, Canada; 6 Department of Population Medicine, Ontario Veterinary College, University of Guelph, Guelph, Ontario, Canada; 7 Canadian Wildlife Health Cooperative, Ontario Veterinary College, University of Guelph, Guelph, Ontario, Canada; University of Kentucky College of Medicine, UNITED STATES

## Abstract

The emergence of the vector *Ixodes scapularis* in Ontario, Canada poses a significant public health risk. Both passive and active surveillance approaches have been employed by public health professionals (i.e., government employees) to monitor for the range expansion of this tick. Field surveillance using drag sampling for questing ticks is a recognized and effective method to identify reproducing tick populations. The degree of effort (i.e., number of visits per site) can enhance the sensitivity and specificity of surveillance, but increased effort conflicts with the cost to public health for field surveillance. Here we developed an indicator to determine the likelihood of *I*. *scapularis* establishment based on field sampling results. Field data from two established populations of *I*. *scapularis* in Ontario were incorporated with previous analyses of surveillance data to create the indicator, which is in the form of a scoring system. The life stage(s) collected, overall abundance and past surveillance findings from a site are all considered and a level is assigned for the likelihood of *I*. *scapularis* establishment based on current field sampling results. The likelihood levels are non-zero (i.e., no *I*. *scapularis* detected, but risk still present due to adventitious ticks), low, medium or high, and recommendations for future surveillance and public health measures are provided. The indicator was validated against field sampling results from five other established sites in the province and correctly categorized all five areas as high likelihood of establishment. The indicator was also applied to field sampling results from 36 sites of unknown status that were visited twice during the period of 2014–2016. There was substantial agreement of levels between measurements, as calculated using a weighted kappa. The indicator can assist public health professionals with the interpretation of field sampling results and direct their efforts for ongoing surveillance and public health interventions for *I*. *scapularis*-borne diseases, including Lyme disease.

## Introduction

In Canada, Lyme disease has been identified as a vector-borne disease of public health importance [1]. The primary causative agent, *Borrelia burgdorferi* sensu stricto, is transmitted by the hard tick *Ixodes scapularis* in central and eastern Canada. Since the early 1990s, there has been notable northward expansion of the tick’s range in the province of Ontario and this has coincided with a dramatic increase in the number of human cases of Lyme disease [2,3]. Range expansion is predicted to continue, in part due to climate change [4,5].

Monitoring this dynamic situation has posed a significant challenge to public health professionals. Passive surveillance, which involves the public submitting ticks collected from themselves (or their pets up to 2007), has been in place in Ontario for decades [6,7]. It provides an effective method to detect areas where risk may be emerging, especially with advanced analyses. However, this approach has low specificity because ticks are deposited annually across Ontario via migratory birds [8]. These ticks, termed adventitious ticks, contribute to a non-zero risk of *I*. *scapularis* across the province and lead to ‘false positive’ sites. Also, the rates of submission of ticks by passive surveillance are heavily influenced by population density [7,9]. Active surveillance is recommended in an area once there has been an increase in tick submissions by passive surveillance, as this finding suggests that there is the presence of an emerging tick population [10]. Active surveillance has high specificity and is useful for determining both the presence of a reproducing population of ticks, and the local infection prevalence of ticks with pathogens [11].

The first field sampling guidelines were outlined in the Canadian Consensus on Lyme Disease [12]. For an area to classified as established for *I*. *scapularis*, all three life stages of the tick need to be detected, either by dragging or on hosts, for two consecutive years. Although this approach is the ‘gold standard’, it is time and labour intensive and has not been feasible given the need for large-scale field sampling [9,10].

Acknowledging these challenges, Ogden and colleagues [10] developed a screening test for active surveillance. This screening test involves conducting tick dragging once per site for three person-hours any time during May to October. If any *I*. *scapularis* are detected, the site is declared a 'risk area', as long as the site is in a known area of *I*. *scapularis* range expansion. Public Health Ontario has adopted this test, but requires *I*. *scapularis* to be detected at a site during both spring and fall drags. The site and surrounding area (20-km radius) is then declared a risk area, and these findings are used to produce a ‘risk map’ for Ontario [13,14].

Defining a risk area provides the first step for assessing the risk of acquiring a tick bite from *I*. *scapularis* (and Lyme disease) within an area. There is, however, limited guidance to further determine the likelihood of *I*. *scapularis* establishment, based on the results of field sampling, in a way that is translated into public health action to appropriately target limited public health resources to the areas of highest risk.

The main objective of our study was to incorporate the known characteristics of *I*. *scapularis* populations into a practical indicator for identifying *I*. *scapularis* establishment (herein referred to as the "indicator") to assist public health professionals with the interpretation and application of findings from field sampling. To collect information on *I*. *scapularis* population dynamics in Ontario, we conducted weekly tick dragging at two established areas from May to October in 2014. These data provided the foundational parameters of the indicator. Additional field sampling was conducted to validate and test the applicability of the indicator.

## Methods

### Field sampling

**Fig 1:**
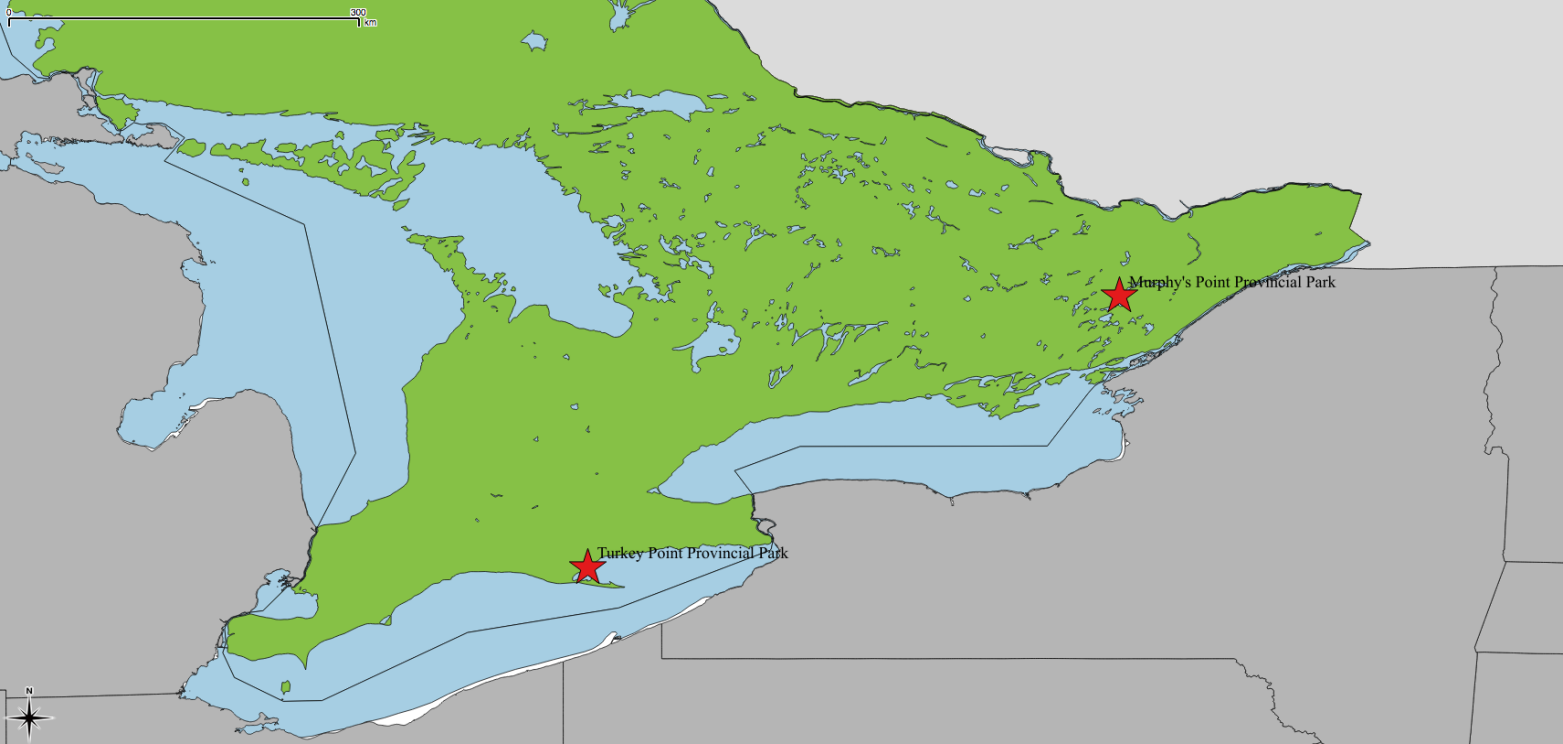
Sites for field sampling. Field sampling was conducted weekly from May to October 2014, alternating between Murphy’s Point Provincial Park and Turkey Point Provincial Park (red stars). Base vector layers were accessed through the Scholars Geoportal at the University of Guelph (http://geo2.scholarsportal.info) from the Ontario Ministry of Natural Resources (province and water) and DMTI Spatial Inc. (Canada and United States of America).

Weekly field sampling was conducted from May to October 2014 (total = 24 weeks), alternating between Turkey Point Provincial Park (TP) (42.704630, -80.334465) and Murphy’s Point Provincial Park (MP) (44.781955, -76.237010) (Fig 1). Both sites have established, reproducing populations of *I*. *scapularis* [15]. TP is located in southern Ontario within an area where *I*. *scapularis* has been present for more than a decade. MP has a recently established population of *I*. *scapularis* and located in eastern Ontario, which is a hot spot for the tick [15, 16]. These sites were purposively chosen to capture some of the differences that may be seen in *I*. *scapularis* populations across the southern and eastern areas of the province due to micro- and macro- climatic and habitat factors.

Tick dragging was conducted at each site visit by dragging a 1 m^2^ flannel drag cloth over the forest and vegetation for three-person hours. The timer was stopped every three minutes to collect ticks from the drag cloth. All life stages of ticks were counted and recorded. Adult and nymphal *I*. *scapularis* were collected and stored in 70% ethanol for laboratory analysis. Permission to conduct field research at the provincial parks was provided by the Parks and Protected Areas Policy Section of the Ontario Ministry of Natural Resources.

### Laboratory analyses

A subset of adult and nymphal *I*. *scapularis* were submitted from each site to the National Microbiology Laboratory at Winnipeg (Public Health Agency of Canada, Winnipeg, Ontario). One hundred and sixty-seven adults of the spring cohort, 58 adults of the fall cohort and 60 nymphs were submitted from TP, while 19 adults of the spring cohort, 99 adults of the fall cohort and 11 nymphs were submitted from MP.

All samples were tested for *B*. *burgdorferi*, *B*. *miyamotoi*, *Anaplasma phagocytophilum* and *Babesia microti*. Laboratory analyses have been previously described [17]. In brief, DNeasy 96 tissue kits were used to extract DNA (QIAGEN Inc. Mississauga, Canada). The 23s ribosomal RNA real-time polymerase chain reaction (PCR) was then used to screen for *Borrelia* spp. If a sample tested positive, it was analyzed with the ospA real-time PCR to detect *B*. *burgdorferi* and the IGS real-time PCR to detect *B*. *miyamotoi*. The glpQ real-time PCR was then used to verify all *B*. *miyamotoi*-positive samples [18]. For *A*. *phagocytophilum*, the msp2 real-time PCR was employed [19], while the real-time PCR for the CCTeta gene was used to detect *B*. *microti* [20]. To verify that contamination did not occur during PCR runs, water blanks were used as negative controls.

### Development of the indicator for determining the likelihood of *I*. *scapularis* establishment

**Fig 2:**
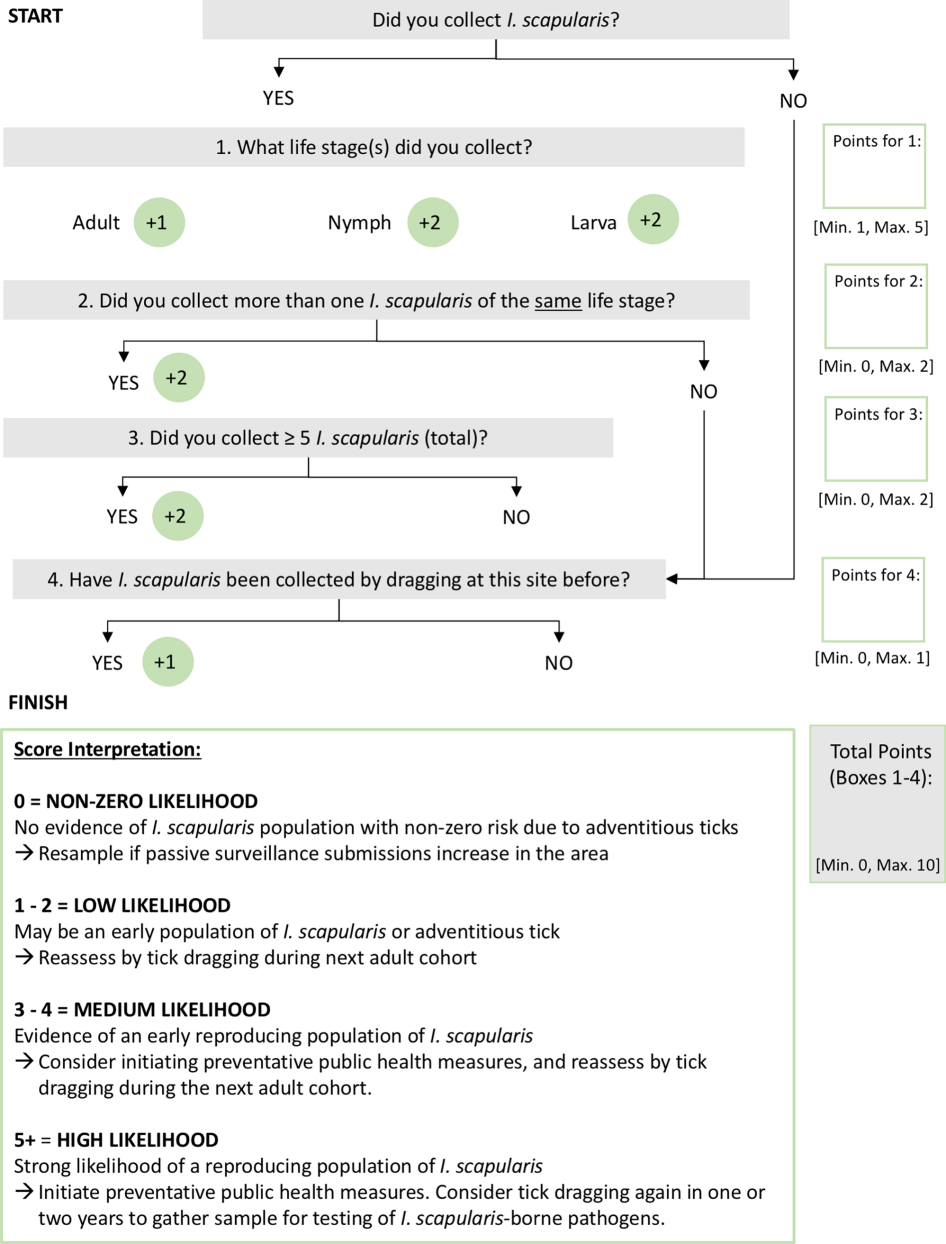
Indicator for determining the likelihood for *I*. *scapularis* establishment. This indicator can be used to assess the likelihood of tick establishment associated with detecting *Ixodes scapularis* during active surveillance at sites in Ontario, Canada. To apply this indicator, begin at the top question marked “START”. Follow the arrows based on the answer to each question, and record the score for each question in the boxes on the right-hand side. Points are allocated based on which criteria are fulfilled, with it being possible to collect points from multiple answers. The minimum and maximum points available from each question are listed under the score box as reference. Once the questions have been completed at “FINISH”, tally the points and match to the corresponding likelihood level.

Both field sampling data and previous research on the dynamics of *I*. *scapularis* populations in Ontario were incorporated into the development of the indicator (Fig 2). Based on the following information, the indicator is divided into criteria on life stage, abundance and previous results of tick dragging.

As in the screening test developed by Ogden and colleagues [10], the detection of one tick of any life stage is used as the basic criterion of potential tick establishment. We expand on this criterion by incorporating the life stage of the tick(s) collected. Most *I*. *scapularis* brought into Ontario via migratory birds are blood fed (i.e., engorged) nymphal ticks [8,21]. These molt into adults and can be collected via tick dragging when they quest. If a questing immature life stage is collected, it is much less likely to be an adventitious tick, and more likely a result of local reproduction. As such, immature ticks are a marker of enhanced likelihood of *I*. *scapularis* establishment [9], and receive additional points in the indicator.

The abundance of ticks collected reflects the density of *I*. *scapularis* in that area and is a key determinant of the probability that a person will be bitten by a tick in that location. When the density is low to medium (i.e., for an emerging population), yield via tick dragging can be low. The total abundance, as well as abundance for each life stage, was added to the indicator to reflect this, and minimum thresholds were established from field sampling results.

Previous results of tick dragging is the final element. Tick dragging can have low sensitivity in areas of low tick density (i.e., an emerging population), leading to false negative sites [10]. Therefore, if the population is just establishing, it is possible to detect ticks sporadically (i.e., at one visit and then not at the next). To acknowledge this limitation, the scoring system considers any previous findings.

The final score is used to determine an overall qualitative measure of the likelihood of *I*. *scapularis* establishment (i.e., non-zero, low, medium and high). Non-zero exists when no ticks are detected at a site. The risk of *I*. *scapularis* cannot be fully negated however, due to the potential introduction of ticks via migratory birds [8,17,21]. Low level corresponds to the basic definition of a risk area; either one tick of any life stage is detected, or a tick has previously been detected by field sampling [10]. Medium level occurs when there is more than one tick, potentially of different life stages. This provides stronger evidence that a reproducing population is present, albeit at low density, and additional monitoring may provide more information. Consideration should be given to communicating potential risk to the public. The highest level reflects strong evidence of a reproducing population of *I*. *scapularis*.

### Indicator performance

The performance of the indicator was tested in two ways. First, the indicator was applied to tick dragging results obtained in the fall of 2013 from five known established sites (as previously determined using the criteria outlined in the Canadian Consensus on Lyme disease). If the indicator performed adequately, these sites should be categorized as high likelihood of *I*. *scapularis* establishment.

Next, the indicator was applied to 36 sites for which there were multiple years of field sampling data (these data were collected contemporaneously for [16,22]). These sites were first visited in either 2014 or 2015. The indicator was applied to the field sampling results and each site was assigned a likelihood level. These sites were revisited in the fall of 2016, and again the indicator was applied to the field sampling results and each site was assigned a likelihood level. Then the kappa statistic was used to determine the degree of agreement of the indicator’s assessment between the findings of the two field samplings [23]. We expect that if the indicator is accurately categorizing risk, then the likelihood level for *I*. *scapularis* establishment at a site should either be the same or one level higher between samplings, since we anticipate there to be continued range expansion of the tick, and/or ongoing establishment of the tick population [4]. Both an unweighted and weighted kappa were calculated [23]. The weighted kappa was chosen to indicate that there was a degree of agreement between adjacent levels, and this decreased with an increase in the difference between likelihood levels. The weight matrix assigned was 1.0 for perfect agreement, 0.8889 for partial agreement (one level removed), 0.5556 for limited agreement (two levels removed) and 0.000 for no agreement (three levels removed). The kappa value was interpreted using the guidelines established by Landis and Koch [24]. STATA version 14.0 (STATACorp, College Station, TX; 2017) was used to calculate the kappa statistics and 95% confidence intervals, with a significance level of α = 0.05.

For all sites used to assess indicator performance, permission to conduct field research was granted by Parks Canada for all national parks, the Ministry of Natural Resources for all provincial parks, designated conservation authorities for each conservation area, local municipal government for county forest tracts and municipal parks, and land owners for all private property.

### Comparison of the indicator and the screening test

We compared the outcomes of the indicator versus the outcomes of the screening test developed by Ogden et al. [10] in terms of public health actions (i.e., additional field sampling at a site, preventative measures). Using the criterion established by Ogden et al. [10], if a one or more *I*. *scapularis* are detected at a site, the site is declared a risk area. No further field sampling is required, and public health interventions should be initiated.

## Results

### Field sampling

**Fig 3:**
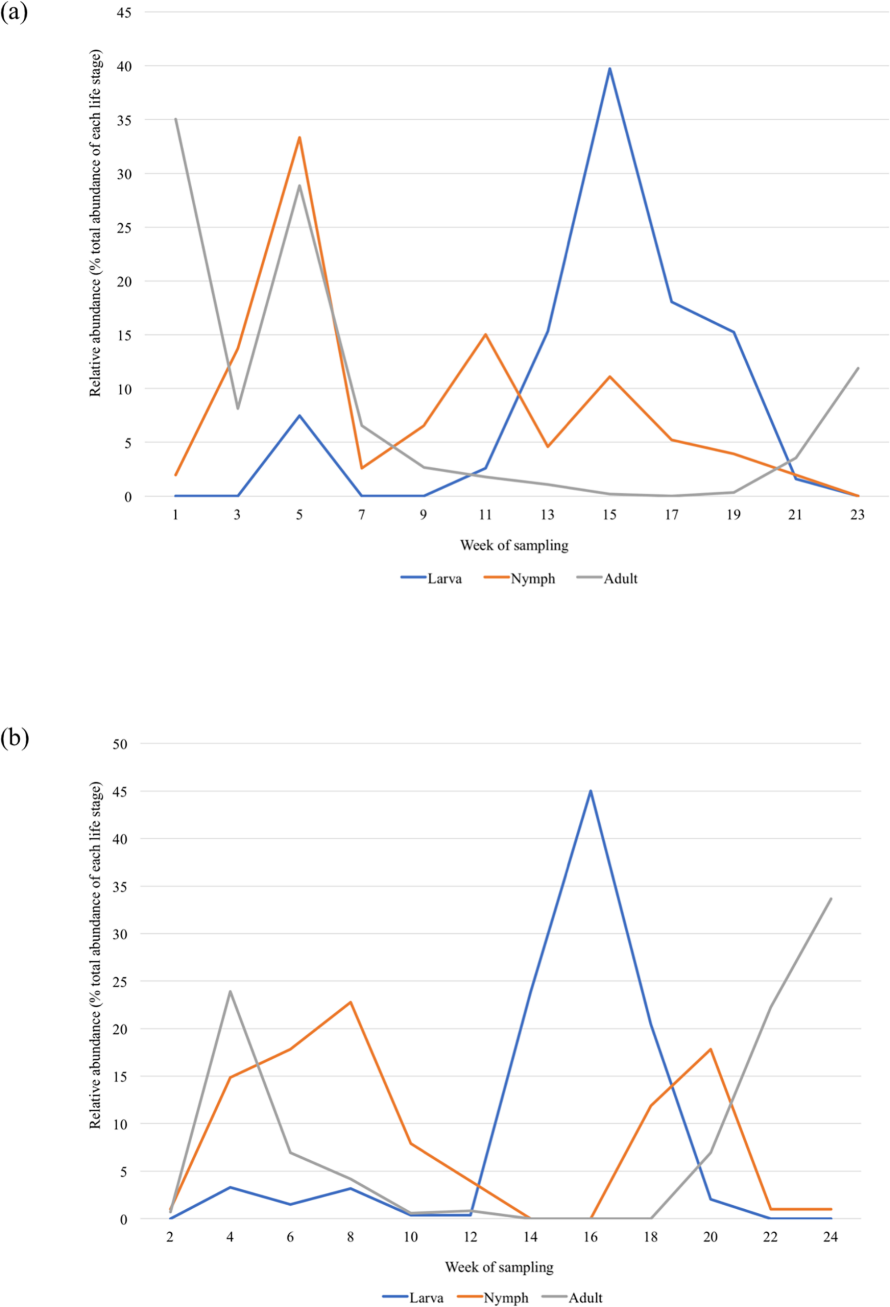
Phenology of *I*. *scapularis* at established sites. The abundance of each *I*. *scapularis* life stage collected varied throughout the timeframe of sampling at both (a) Turkey Point Provincial Park and (b) Murphy’s Point Provincial Park in Ontario. Adult *I*. *scapularis* (green line) demonstrated a bimodal peak in the early spring and fall. Nymphal *I*. *scapularis* (orange line) were active during late spring to early summer. Larval *I*. *scapularis* (blue line) were active predominately in late summer, but also had a small peak of activity in late spring. Weekly abundance is presented as the percentage of the total abundance of each life stage collected over the sampling period. The weeks of sampling correspond to each month: 1–3 = May, 4–8 = June, 9–12 = July, 13–16 = August, 17–20 = September, and 21–24 = October.

*Ixodes scapularis* were collected at each site (i.e., TP or MP) every week throughout the sampling season (S1 Table). The median number of ticks collected during each sampling period (i.e., three-person hours) was 134, with a minimum of 6 and a maximum of 756 (Table 1). The data are right-skewed due to the high abundance of the larval life stage. There were large seasonal variations in the numbers of ticks collected at the two established sites (Fig 3). However, one life stage of tick, with multiple individuals of that life stage, was always found, and the total tick abundance was always greater than five ticks (Table 1).

**Table 1:** *Ixodes scapularis* collected via field sampling. The median and range of each life stage of *I*. *scapularis* collected after three person-hours of tick dragging from May to October 2014 at Turkey Point Provincial Park and Murphy’s Point Provincial Park.

Life stage	Turkey Point Provincial Park Median (range)	Murphy’s Point Provincial Park Median (range)
Larva	25 (0–477)	30 (0–756)
Nymph	8 (0–51)	6 (0–23)
Adult	18 (0–198)	18 (0–242)
Total	129 (25–495)	134 (6–756)

### Laboratory analyses

The *B*. *burgdorferi* infection prevalence of the spring 2014 and fall 2014 adult cohorts at TP were 30.0% (95% confidence interval 20.4%-34.2%) and 31.0% (19.5%-44.5%), respectively, while the nymphal infection prevalence was 11.6% (4.8%-22.6%). At MP, the infection prevalence of the spring 2014 and fall 2014 cohorts were 36.8% (16.3%-61.6%) and 78.7% (69.4%-86.4%), respectively. The nymphal infection prevalence was 18.2% (2.3%-51.8%). Three *I*. *scapularis* from MP were positive for *B*. *miyamotoi* (2.3% (0.5%-6.6%)), one of which was co-infected with *B*. *burgdorferi*. All samples were negative for *A*. *phagocytophilum* and *B*. *microti*.

### Indicator performance

The indicator assessed all five established sites as high likelihood, based on the field sampling results collected in the fall of 2013 (Table 2).

**Table 2:** Application of indicator. The scores based on the application of the indicator for five established sites in Ontario based on tick dragging in the summer and fall of 2013.

Site	Field sampling results	1. What life stage(s) did you collect?	2. Did you collect ≥ 2 *I*. *scapularis* of the same life stage?	3. Did you collect ≥ 5 *I*. *scapularis* (total)?	4. Have *I*. *scapularis* been collected at this site before?	Total score and likelihood level
Hill Island^1^ (44.353838, -75.967772)	16 nymphs, 238 larvae	nymph = 2 points, larva = 2 points	yes = 2 points	yes = 2 points	yes = 1 point	9 = high
Thwartway Island^1^ (44.29416, -76.15020)	9 nymphs, 478 larvae	nymph = 2 points, larva = 2 points	yes = 2 points	yes = 2 points	yes = 1 point	9 = high
Camelot Island^1^ (44.30168, -76.11152)	2 nymphs, 223 larvae	nymph = 2 points, larva = 2 points	yes = 2 points	yes = 2 points	yes = 1 point	9 = high
Long Point Provincial Park (42.58166, -80.39514)	1 adult, 25 larvae	adult = 1 point, larva = 2 points	yes = 2 points	yes = 2 points	yes = 1 point	8 = high
Rondeau Provincial Park (42.31740, -81.84723)	58 adults	adult = 1 point	yes = 2 points	yes = 2 points	yes = 1 point	6 = high

^1^Part of Thousand Islands National Park

For the field sites of unknown status, the indicator assessed 21 as non-zero, 4 as low, 5 as medium and 6 as high likelihood of *I*. *scapularis* establishment following field sampling in 2014 and 2015. When the indicator was reapplied to assess these sites, based on follow-up field sampling in 2016, 28 sites stayed the same level, 3 increased by one level, 2 increased by two levels and 1 increased by three levels. Only two sites decreased in risk, 1 by one level and the other by two levels (S2 Table). The unweighted kappa was 0.67 (95% CI 0.58–0.75) (p<0.001) and the weighted kappa was 0.74 (95% CI 0.74–0.88) (p<0.001), which is within the range of values supportive of substantial agreement [24].

### Comparison of the indicator and the screening test

The recommended public health actions following each field sampling time frame using the screening test by Ogden et al. [10] and our indicator were compared (Table 3). The recommendation to repeat field sampling was potentially higher with our indicator, while the recommendation to begin preventative measures was less frequent. This is because each site had to meet the criteria of medium or high likelihood to qualify for this recommendation.

**Table 3:** The recommendation for public health actions following each field sampling timeframe based on the screening test outlined by Ogden et al. [10] and our indicator.

Approach	Field sampling 2014–2015	Field sampling 2016	Outcome comparison
**Screening test by Ogden et al. [10]**	Risk area = 15 of 36 sites • Resampling would not be recommended at 15 risk areas • Resampling could be conducted at 21 sites without *I*. *scapularis*	Risk area = 5 of 21 sites resampled • Resampling would not be recommended at 5 new risk areas • Resampling could be conducted at 16 sites without *I*. *scapularis*	• Decreased frequency of resampling • Potential increased requirements for public health interventions at all risk areas • Of the sites that would not have been resampled, 3 were negative at the second sampling
**Indicator for determining the likelihood of *I*. *scapularis* establishment**	Non-zero = 21 sites, Low = 4 sites, Medium = 5 sites, High = 6 • Resampling would be recommended for a minimum of 9 sites (low and medium) • Public health interventions would be recommended for 11 sites (medium and high)	Non-zero = 16 sites, Low = 7 sites, Medium = 7 sites, High = 6 • Resampling would be recommended for a minimum of 14 sites (low and medium) • Public health interventions would be recommended for 13 sites (medium and high)	• Potentially increased frequency of resampling • More stringent requirements for public health interventions • Greater information gathered on tick population

## Discussion

The ongoing invasion of *I*. *scapularis* into Ontario, Canada has posed many challenges for public health officials; conducting active field surveillance to determine the geographic risk of the tick and associated pathogens is one major challenge. In this study, we developed and validated an indicator to assist public health officials with the interpretation of field sampling results to determine the likelihood of a reproducing population of *I*. *scapularis*.

Application of the indicator would be straight-forward. The standardized field sampling approach, which was previously outlined in Ogden et al. [10], is simple to use. Field sampling can be conducted any time during the active season of *I*. *scapularis*, which is generally between May to November in eastern Canada [25,26]. During the known active season, there is a bimodal peak in adult tick numbers in the spring and fall and a nymphal peak in early summer. There are two larval peaks, with a small increase in activity in spring and then the largest period of activity in late summer [27,28]. This is supported by our findings at TP and MP, which illustrate the general pattern, as well as the potential variations that may be seen at different sites across Ontario due to macro and microclimatic and habitat factors. Daily and diurnal fluctuations in temperature and humidity impact tick activity [29,30], while excessively hot temperatures (>30°C) reduce the tick’s activity [31]. Other variations in the numbers of collected ticks can occur for several reasons, which may be associated with methodology. The distribution of *I*. *scapularis* in the environment can be highly variable, and therefore tick dragging needs to be conducted over a representative area of each study site [32,33]. If a drag cloth becomes wet (either from rain or dew), the ticks are less likely to attach [13]. Furthermore, where site vegetation is particularly dense, ticks can be dislodged from the drag, so it may be necessary to check the cloth more frequently.

There are several other considerations for tick dragging. Selected sites need to be ecologically suitable. *Ixodes scapularis* are found in wooded and brushy areas, and sites for field surveillance should be selected accordingly [26,34–36]. If follow-up field sampling is indicated, it is ideal to sample for a subsequent cohort of ticks, as evidence of a new cohort represents successful development and reproduction of the population. In eastern Canada, adults and larvae that are active in the spring are most likely ticks that did not feed successfully in the previous year and overwintered unfed [25]. Therefore, if sampling is conducted in the spring for adults, any adults collected in follow-up sampling will be of a different cohort, while adults collected in the fall and the subsequent spring will most likely be of the same cohort.

When the screening test developed by Ogden et al. [10] and our indicator were compared, the main difference was the number of sites at which the public health actions (i.e., additional field sampling and preventative interventions) would be warranted. Limited resampling is indicated using Ogden et al. [10], while our indicator encourages additional field sampling for sites classified as low or medium likelihood. Preventative interventions are suggested as soon as an area is classified as a risk area, while our approach suggests preventative interventions for medium and high likelihood sites. This may result in economies in public health expenditure on interventions that outweigh the costs of revisiting sites at which only one tick was found. Tick dragging can be completed with minimal financial investment ($100 - $200 CND/site), but is highly dependent on which staff conduct the field sampling (i.e., summer student versus public health nurse or public health inspector), the amount of travel required to access a site, and the amount of supplies consumed (personal communication with Public Health Units). Further cost-benefit studies would be of value. It is important to note that the purpose of the indicator is not to replace the screening test. The screening test plays a valuable role in the production of the risk map, which allows for clear communication of the potential risk of *I*. *scapularis* to the public. Our indicator provides more detailed assessment to assist public health professionals with ongoing field sampling and targeted interventions.

An important point in question is the frequency of resampling. Our indicator recommends resampling at the next cohort at sites classified as low and medium likelihood. Tick abundance usually increases over several years in newly-established populations [37]. Early tick populations may also die out by stochastic fade out, or due to low tick densities may not always be detectable by drag sampling [21,38]. Under these circumstances, a longer resampling time frame may be appropriate, especially for areas deemed low likelihood.

Public health surveillance for *I*. *scapularis* is conducted to determine the risk of disease due to *I*. *scapularis*-borne pathogens. However, our indicator does not consider the presence of pathogens and this was purposively excluded. In eastern Canada, *I*. *scapularis* populations generally develop free of *B*. *burgdorferi*; current research estimates an approximate five-year lag between tick population establishment and the establishment of *B*. *burgdorferi* transmission cycles [21,37]. On the other hand, adventitious ticks can carry *B*. *burgdorferi*, and based on passive surveillance findings 13 to 15% of ticks are infected [8]. Therefore, as in [10], the detection of *B*. *burgdorferi* was not used as a marker for the likelihood of tick establishment and not incorporated into the indicator. At a regional, multi-site scale, laboratory testing is an essential component of public health surveillance because the presence of pathogens can be identified along with the infection prevalence.

There are several limitations that should be acknowledged. First, although tick dragging is a highly valuable strategy for surveillance, it can have low sensitivity in areas of low tick density, which leads to an increase in false negative sites [10]. However, in that context, the risk to the public is very low anyways. For the development of the indicator, only two sites were used over one season. This may limit our ability to detect greater intra- and inter-annual variations in tick populations. For validation of the indicator, we had a small sample size of established sites. Finally, it is important to note that this indicator was developed using data from Ontario and is most suitable for application in Ontario, and potentially other eastern provinces. The population dynamics of *I*. *scapularis* are different in central Canada and therefore this indicator should be validated with data from the geographic areas of interest prior to implementation [28,37].

Our study has provided an indicator for public health professionals to use when conducting field sampling for *I*. *scapularis*. With this indicator, the likelihood of a reproducing population of ticks can be quickly and easily assessed with one site visit, and public health preventative measures for Lyme disease and other *I*. *scapularis*-borne pathogens can be initiated in the areas of highest risk.

Now the indicator needs to be field-tested with public health professionals. This will ensure it is easy to use by the audience for which it is designed. Qualitative feedback via focus groups and surveys could be of benefit to further refine the indicator. It may also be beneficial to compare the indicator with other outcomes, such as the density of infected nymphs, which are commonly used measures for risk assessment.

## Supporting information

S1 Table Tick dragging results. The abundance of each life stage of *I*. *scapularis* collected via tick dragging during each visit to Turkey Point Provincial Park and Murphy’s Point Provincial Park from May to October 2014.
(DOCX)

S2 Table Application of indicator. The scores based on the application of the indicator for sites of unknown status sampled during 2014–2015 and again in 2016 (NZ = non-zero, L = low, M = medium, H = high).
(DOCX)
