# Supplementary material for: A field-based indicator for determining the likelihood of Ixodes scapularis establishment at sites in Ontario, Canada
Source: PLoS One. 2018 Feb 27;13(2):e0193524. doi: 10.1371/journal.pone.0193524 (PMC5828431; doi:10.1371/journal.pone.0193524)
Supplement: S1 Table — The abundance of each life stage of I. scapularis collected via tick dragging during each visit to Turkey Point Provincial Park and Murphy’s Point Provincial Park from May to October 2014. (DOCX) [file pone.0193524.s001.docx]

| **Week of sampling** | **Date** | **Provincial Park** | **Abundance** | | | | | |
| --- | --- | --- | --- | --- | --- | --- | --- | --- |
|  |  |  | **Male** | **Female** | **Adult Total** | **Nymph** | **Larvae** | **Total** |
| 1 | 2014-05-04 | Turkey Point | 97 | 101 | 198 | 3 | 0 | 201 |
| 2 | 2014-05-20 | Murphy’s Point | 3 | 2 | 5 | 1 | 0 | 6 |
| 3 | 2014-05-26 | Turkey Point | 21 | 25 | 46 | 21 | 0 | 67 |
| 4 | 2014-06-04 | Murphy’s Point | 94 | 78 | 172 | 15 | 55 | 242 |
| 5 | 2014-06-09 | Turkey Point | 98 | 65 | 163 | 51 | 90 | 304 |
| 6 | 2014-06-17 | Murphy’s Point | 25 | 25 | 50 | 18 | 25 | 93 |
| 7 | 2014-06-27 | Turkey Point | 13 | 24 | 37 | 4 | 0 | 41 |
| 8 | 2014-06-28 | Murphy’s Point | 17 | 13 | 30 | 23 | 53 | 106 |
| 9 | 2014-07-09 | Turkey Point | 7 | 8 | 15 | 10 | 0 | 25 |
| 10 | 2014-07-17 | Murphy’s Point | 1 | 3 | 4 | 8 | 6 | 18 |
| 11 | 2014-07-23 | Turkey Point | 3 | 7 | 10 | 23 | 31 | 64 |
| 12 | 2014-07-29 | Murphy’s Point | 3 | 3 | 6 | 4 | 6 | 16 |
| 13 | 2014-08-07 | Turkey Point | 3 | 3 | 6 | 7 | 184 | 197 |
| 14 | 2014-08-11 | Murphy’s Point | 0 | 0 | 0 | 0 | 401 | 401 |
| 15 | 2014-08-22 | Turkey Point | 1 | 0 | 1 | 17 | 477 | 495 |
| 16 | 2014-08-26 | Murphy’s Point | 0 | 0 | 0 | 0 | 756 | 756 |
| 17 | 2014-09-04 | Turkey Point | 0 | 0 | 0 | 8 | 217 | 225 |
| 18 | 2014-09-10 | Murphy’s Point | 0 | 0 | 0 | 12 | 343 | 355 |
| 19 | 2014-09-15 | Turkey Point | 1 | 1 | 2 | 6 | 183 | 191 |
| 20 | 2014-09-26 | Murphy’s Point | 25 | 25 | 50 | 18 | 34 | 102 |
| 21 | 2014-10-01 | Turkey Point | 8 | 12 | 20 | 3 | 19 | 42 |
| 22 | 2014-10-09 | Murphy’s Point | 85 | 75 | 160 | 1 | 0 | 161 |
| 23 | 2014-10-14 | Turkey Point | 37 | 30 | 67 | 0 | 0 | 67 |
| 24 | 2014-10-19 | Murphy’s Point | 102 | 140 | 242 | 1 | 0 | 243 |
