# Supplementary material for: A field-based indicator for determining the likelihood of Ixodes scapularis establishment at sites in Ontario, Canada
Source: PLoS One. 2018 Feb 27;13(2):e0193524. doi: 10.1371/journal.pone.0193524 (PMC5828431; doi:10.1371/journal.pone.0193524)
Supplement: S2 Table — The scores based on the application of the indicator for sites of unknown status sampled during 2014–2015 and again in 2016 (NZ = non-zero, L = low, M = medium, H = high). (DOCX) [file pone.0193524.s002.docx]

| **Site ID** | **Geographic coordinates** | **Field sampling 2014-2015** | | | | | **Field sampling 2016** | | | | | **Change in likelihood level** |
| --- | --- | --- | --- | --- | --- | --- | --- | --- | --- | --- | --- | --- |
|  |  | **Adult** | **Nymph** | **Larvae** | **Score** | **Risk Level** | **Adult** | **Nymph** | **Larvae** | **Score** | **Risk Level** |  |
| 1 | 44.6009, -75.87157 | 4 | 0 | 0 | 3 | M | 2 | 0 | 0 | 4 | M | No change |
| 2 | 42.66227, -81.8115 | 1 | 0 | 0 | 1 | L | 1 | 0 | 0 | 2 | L | No change |
| 4 | 43.22347, -81.87037 | 1 | 0 | 0 | 1 | L | 1 | 0 | 0 | 2 | L | No change |
| 5 | 44.27665, -77.34817 | 0 | 2 | 0 | 4 | M | 1 | 1 | 0 | 4 | M | No change |
| 13 | 43.61236, -80.25098 | 0 | 0 | 0 | 0 | NZ | 0 | 0 | 0 | 0 | NZ | No change |
| 21 | 45.11592, -76.08365 | 0 | 0 | 0 | 0 | NZ | 1 | 0 | 0 | 1 | L | Increase by one |
| 22 | 45.24577, -76.25332 | 0 | 0 | 0 | 0 | NZ | 1 | 0 | 0 | 1 | L | Increase by one |
| 24 | 45.02937, -75.65957 | 0 | 0 | 0 | 0 | NZ | 4 | 3 | 0 | 7 | H | Increase by three |
| 25 | 44.85191, -75.62514 | 0 | 0 | 0 | 0 | NZ | 0 | 2 | 0 | 4 | M | Increase by two |
| 26 | 44.76123, -75.4823 | 2 | 0 | 0 | 3 | M | 2 | 0 | 0 | 4 | M | No change |
| 48 | 43.93148, -80.08172 | 0 | 0 | 0 | 0 | NZ | 0 | 0 | 0 | 0 | NZ | No change |
| 49 | 44.68426, -76.39207 | 0 | 3 | 15 | 8 | H | 0 | 0 | 0 | 1 | L | Decrease by two |
| 54 | 44.9937, -76.5449 | 0 | 1 | 0 | 2 | L | 0 | 0 | 0 | 1 | L | No change |
| 66 | 44.7241, -76.7241 | 0 | 2 | 2 | 6 | H | 9 | 0 | 30 | 8 | H | No change |
| 67 | 44.55852, -76.78022 | 0 | 2 | 0 | 4 | M | 1 | 1 | 80 | 10 | H | Increase by one |
| 69 | 44.76713, -77.87651 | 0 | 0 | 0 | 0 | NZ | 0 | 0 | 0 | 0 | NZ | No change |
| 88 | 44.39167, -77.31478 | 0 | 0 | 0 | 0 | NZ | 0 | 0 | 0 | 0 | NZ | No change |
| 92 | 45.03079, -74.79646 | 0 | 3 | 9 | 8 | H | 10 | 1 | 21 | 10 | H | No change |
| 95 | 45.01379, -74.77392 | 0 | 1 | 10 | 8 | H | 2 | 0 | 43 | 8 | H | No change |
| 101 | 44.90105, -77.20445 | 0 | 0 | 0 | 0 | NZ | 0 | 0 | 0 | 0 | NZ | No change |
| 105 | 42.81278, -80.62072 | 1 | 0 | 0 | 1 | L | 0 | 0 | 0 | 1 | L | No change |
| 115 | 43.43203, -80.13249 | 0 | 0 | 0 | 0 | NZ | 0 | 0 | 0 | 0 | NZ | No change |
| 116 | 43.50983, -80.22028 | 0 | 0 | 0 | 0 | NZ | 0 | 0 | 0 | 0 | NZ | No change |
| 117 | 43.54911, -80.18226 | 0 | 0 | 0 | 0 | NZ | 0 | 0 | 0 | 0 | NZ | No change |
| 119 | 43.9618, -80.40017 | 0 | 0 | 0 | 0 | NZ | 0 | 0 | 0 | 0 | NZ | No change |
| 120 | 43.87245, -78.79458 | 1 | 0 | 1 | 3 | M | 2 | 0 | 0 | 4 | M | No change |
| 122 | 44.61987, -78.04642 | 0 | 2 | 2 | 6 | H | 3 | 0 | 0 | 4 | M | Decrease by one |
| 128 | 44.5606, -77.11739 | 3 | 0 | 3 | 7 | H | 6 | 1 | 80 | 10 | H | No change |
| 129 | 44.83796, -77.67705 | 0 | 0 | 0 | 0 | NZ | 0 | 0 | 0 | 0 | NZ | No change |
| 139 | 45.46495, -76.695 | 0 | 0 | 0 | 0 | NZ | 2 | 0 | 0 | 3 | M | Increase by two |
| 149 | 45.63326, -77.0672 | 0 | 0 | 0 | 0 | NZ | 0 | 0 | 0 | 0 | NZ | No change |
| 150 | 45.69362, -76.83303 | 0 | 0 | 0 | 0 | NZ | 0 | 0 | 0 | 0 | NZ | No change |
| 151 | 45.35601, -76.8116 | 0 | 0 | 0 | 0 | NZ | 0 | 0 | 0 | 0 | NZ | No change |
| 152 | 45.11511, -77.11427 | 0 | 0 | 0 | 0 | NZ | 0 | 0 | 0 | 0 | NZ | No change |
| 153 | 45.45315, -77.47802 | 0 | 0 | 0 | 0 | NZ | 0 | 0 | 0 | 0 | NZ | No change |
| 154 | 45.18743, -77.5347 | 0 | 0 | 0 | 0 | NZ | 0 | 0 | 0 | 0 | NZ | No change |
